# Multimodal assessment of NIBS-induced adaptive plasticity in aging prefrontal networks: ERP and cognitive evidence

**DOI:** 10.3389/fnmol.2026.1954473

**Published:** 2026-10-01

**Authors:** Davide Crivelli, Michela Balconi

**Affiliations:** 1 International Research Center for Cognitive Applied Neuroscience (IrcCAN), Università Cattolica del Sacro Cuore, Milan, Italy; 2 Faculty of Psychology, Università Cattolica del Sacro Cuore, Milan, Italy

**Keywords:** ERP, healthy aging, neuroassessment, neuroempowerment, NIBS, tDCS

## Abstract

Non-invasive brain stimulation may enhance age-sensitive executive functions by modulating task-engaged prefrontal networks, but behavioral effects remain heterogeneous and their physiological specificity is poorly understood. This pre-post study examined neuropsychological and electrophysiological responses to cognitive training paired with active or sham transcranial direct current stimulation (tDCS) over the dorsolateral prefrontal cortex. Thirty-three older adults were randomly assigned to active tDCS plus cognitive training, sham tDCS plus cognitive training, or wait-list control. Training groups completed 24 sessions across 8 weeks. Before and after intervention, participants underwent neuropsychological assessment and EEG recording during a Stroop-like task; N2 amplitude and latency were extracted at F3, Fz, and F4. Linear mixed-effects models tested Group × Time effects. Significant interactions emerged for non-verbal executive functions and associative fluency, with improvements confined to the active-tDCS group. Other cognitive measures improved over time without group-specific effects. Active tDCS selectively increased N2 amplitude in correspondence to the stimulated site, whereas no comparable change occurred after sham stimulation or in controls. These findings suggest that repeated right-prefrontal tDCS may specifically support higher-order executive functions and induce selective modulation near the stimulation site. Physiological measures may help clarify the anatomical and functional specificity of NIBS-related modulation.

## 1 Introduction

Healthy aging is accompanied by marked interindividual variability in cognitive functioning. Executive functions are particularly vulnerable because they depend on prefrontal systems that undergo substantial structural and functional change in later life (Cabeza and Dennis, 2013; Kennedy and Raz, 2009; Raz et al., 2005). Declines in inhibitory control, cognitive flexibility, monitoring, and attentional allocation may affect the management of complex everyday activities, making executive functioning a central target for preventive healthcare and neuroempowerment.

Age-related cognitive change, however, is not reducible to progressive loss. Older adults may preserve performance through altered recruitment of neural resources. The HAROLD (Hemispheric Asymmetry Reduction in Older adults) and PASA (Posterior-Anterior Shift in Aging) models describe reduced hemispheric asymmetry and greater prefrontal recruitment during demanding tasks (Cabeza, 2002; Davis et al., 2008). Such patterns may reflect compensation, although increased activation is functionally adaptive only when it contributes to maintained or improved performance (Cabeza et al., 2018). This distinction is central to non-invasive brain stimulation (NIBS), because stimulation-induced changes may foster adaptive plasticity or, under suboptimal conditions, reinforce inefficient network dynamics.

Cognitive training can engage residual plasticity and improve trained abilities in healthy older adults, but transfer across cognitive domains remains variable (Kelly et al., 2014; Lampit et al., 2014; Siegert et al., 2021). Combining training with NIBS may strengthen activity-dependent plasticity by modulating the responsivity of networks that are concurrently engaged. Transcranial direct current stimulation (tDCS) applies weak electrical currents to alter cortical excitability and network-level processing, and the dorsolateral prefrontal cortex is considered a relevant target for neuroempowerment because of its role in executive attention, monitoring, relational integration, and flexible control (Nitsche and Paulus, 2011; Santarnecchi et al., 2015; Shin et al., 2015; Tatti et al., 2016). Yet findings in healthy older adults remain heterogeneous: some studies report gains after repeated prefrontal stimulation, particularly when paired with cognitive training, whereas others find limited or no advantage over sham or control conditions (Antal et al., 2017; Huo et al., 2018; Indahlastari et al., 2021; Nilsson et al., 2017; Park et al., 2014; Tatti et al., 2016).

A major challenge is therefore to identify neurobiological markers that distinguish adaptive from nonspecific responses to stimulation. Most studies rely primarily on behavioral outcomes, which provide limited information about the temporal dynamics of intervention-related network change. Event-related potentials can help address this gap by offering millisecond-resolution indices of cortical information processing. The frontocentral N2, typically observed approximately 200–350 ms after stimulus onset, has been linked to conflict detection, mismatch processing, response selection, and cognitive-control recruitment (Folstein and Van Petten, 2008; Larson et al., 2014). Aging may alter its amplitude, latency, and topography, whereas executive training may modify the organization of prefrontal N2 activity (Lucci et al., 2013; Najberg et al., 2021; Wild-Wall et al., 2008).

The present pilot study used a multimodal pre-post design to examine neuropsychological and electrophysiological responses to individualized cognitive training paired with sham stimulation, the same training combined with repeated anodal tDCS over the right prefrontal cortex, and a wait-list condition. By simultaneously modeling the three groups, the study examined whether pre-to-post changes differed across conditions and whether active tDCS was associated with selective changes in executive functioning and frontal N2 activity beyond general time-related or training-related effects. We expected both cognitively trained groups to improve on selected executive-attentional measures, with more pronounced changes in participants receiving active tDCS for functions supported by right-prefrontal networks. While explorative, we also hypothesized active stimulation to produce selective N2 ERP (Event Related Potential) modulation.

## 2 Materials and methods

### 2.1 Participants and study design

Thirty-three community-dwelling older adults were recruited through local elderly associations and randomly assigned via extraction to a wait-list control group (WL; *n* = 12; 8 women; age *M* = 70.50, SD = 7.33; education *M* = 13.50, SD = 3.37), a sham-tDCS plus cognitive-training group (sham-Cog; *n* = 10; 8 women; age *M* = 72.40, SD = 4.84; education *M* = 10.10, SD = 3.18), or an active-tDCS plus cognitive-training group (tDCS-Cog; *n* = 11; 7 women; age *M* = 74.73, SD = 6.66; education *M* = 9.73, SD = 4.00). Participants had normal or corrected-to-normal vision and no neuropsychological impairment. Cognitive-health eligibility was established through neuropsychological screening (including Mini Mental State Examination, Geriatric Depression Scale, and Instrumental Activities of Daily Living scale) and anamnestic interview. Further exclusion criteria included organ transplantation, brain surgery, and history of neurological or psychiatric disease. Participants in the stimulation conditions also completed a tDCS safety screening.

The pilot study used a three-arm controlled pre-post design comprising an active-stimulation condition, a sham-stimulation condition, and a wait-list control condition. Both stimulation groups completed individualized cognitive-training and followed the same 8-week schedule of three sessions per week. Sham stimulation controlled for procedural and expectancy effects. The wait-list condition provides an additional reference for changes associated with repeated assessment and the passage of time, yet does not control for nonspecific intervention-related factors and contextual factors. Assessments were conducted at baseline (t0) and immediately post-intervention (t1). Assessors administering cognitive tests and EEG analysts were blinded to participant’s condition. The protocol (ID: protD322013) was approved by the Ethics Committee of the Department of Psychology, Università Cattolica del Sacro Cuore, Milan. All participants provided written informed consent, and procedures conformed to the Declaration of Helsinki.

### 2.2 Assessment procedure and instruments

At each time point, participants completed standardized measures of global cognition, executive functions, attention, and memory. The battery included the Stroop test (Caffarra et al., 2002), Raven’s Progressive Matrices (Bianchi and Dai Prà, 2008; Spinnler and Tognoni, 1987), phonemic, semantic, and associative verbal-fluency tests (Bianchi and Dai Prà, 2008; Novelli et al., 1986; Spinnler and Tognoni, 1987), Attention Matrices (Bianchi and Dai Prà, 2008; Spinnler and Tognoni, 1987), Trail Making Test (Giovagnoli et al., 1996), disyllabic span (Bianchi and Dai Prà, 2008; Monaco et al., 2013; Spinnler and Tognoni, 1987), Corsi block-tapping test (Bianchi and Dai Prà, 2008; Spinnler and Tognoni, 1987), Rey Auditory Verbal Learning Test (Carlesimo et al., 1996), and a visuospatial supra-span learning task (Bianchi and Dai Prà, 2008; Spinnler and Tognoni, 1987). Age-, education-, and sex-corrected performance scores were used when available.

Participants also performed a 120-trial computerized Stroop-like task. Congruent and incongruent color-word stimuli (respectively, 25% and 75% of trials, based on color-word combinations) were presented for 300 ms, and participants classified each stimulus as congruent, such as the word “RED” displayed in red, or incongruent, such as the word “RED” displayed in blue. The task provides overall accuracy and omission scores across trials, such overall scores were collected as indices of attentional-regulation efficacy. During task execution, electroencephalographic activity was recorded to extract event-related potentials and derive electrophysiological markers of attentional regulation and processing efficiency (Crivelli and Balconi, 2017).

EEG was acquired using a 15-channel V-Amp recording system (Brain Products GmbH, Gilching, Germany) with Ag/AgCl electrodes positioned according to the 10–20 International System and referenced to linked earlobes (Sampling Rate = 1,000 Hz, electrode impedance threshold = 5 kΩ). Vertical electro-oculographic activity was recorded concurrently to identify ocular artifacts for subsequent correction and rejection. EEG data were filtered offline using a 0.1–30 Hz band-pass filter with a 24 dB/octave slope and processed using a semi-automated Independent Component Analysis-based artifact-correction procedure (Jung et al., 2000). Following semi-automated artifact correction, the continuous signal was segmented relative to stimulus onset into 1000-ms epochs, including a 200-ms prestimulus baseline. Epochs were then visually inspected to remove residual ocular, muscular, and movement-related artifacts. Artifact-free segments were averaged separately for congruent and incongruent trials to obtain condition-specific ERP waveforms (individual rejection rate range: 5%–20%). Morphological inspection of the averaged waveforms consistently identified a negative deflection peaking at approximately 200 ms, classified as the N2 component. N2 peak amplitude and latency were extracted at F3, Fz, and F4 using a semi-automatic weighted peak-detection algorithm based on a fixed time window (in our case, 150–300 ms) implemented in the signal processing software (Brain Vision Analyser, Brain Products GmbH, Gilching, Germany).

### 2.3 Training procedures

Both stimulation groups completed the same computerized cognitive-training program using ERICA software (Giunti OS, Florence, Italy). Given the applicative nature of the study, the program was designed to preserve ecological relevance by allowing individualization of task difficulty while maintaining a standardized training structure. Training frequency and duration were constant across participants, with each session lasting approximately 45–60 min. The time devoted to cognitive exercises and the number and type of tasks administered within each session were kept constant across participants, covering attention, memory, and verbal and non-verbal executive functions. Individualization concerned the level of task difficulty and its progression according to ongoing performance. Specifically, task difficulty was increased when participants achieved fewer than 20% errors in the preceding sessions, providing a systematic criterion for progression while allowing the level of challenge to be adapted to individual performance. Thus, participants received a comparable overall training dose and task structure, while the difficulty level was progressively tailored to their individual performance.

Transcranial direct current stimulation was administered using a BrainStim device (EMS srl, Bologna, Italy). In the active condition, a continuous 1.5-mA anodal current was delivered for 15 min using 35-cm^2^ conductive rubber electrodes placed inside saline-soaked sponge pockets. Cognitive exercises began 5 min after stimulation onset. Following the International 10–20 System, the anode was positioned over F4 to target the right prefrontal cortex, whereas the cathode was positioned over the contralateral supraorbital site Fp1, resulting in a cephalic bipolar montage. The resulting nominal current density was approximately 0.043 mA/cm^2^. Sessions were administered three times per week for 8 weeks, for a planned total of 24 stimulation sessions. All participants took part and completed all planned sessions.

Participants in sham-Cog underwent sham stimulation in addition to the same cognitive-training program and session schedule. Sham stimulation reproduced the device setup and initial cutaneous sensations of active stimulation without sustained neuromodulation. As in the active condition, the procedure included 30-s ramp-up and 30-s ramp-down phases. The WL group continued its usual activities and received neither stimulation nor structured cognitive training.

### 2.4 Data analysis

Neuropsychological, computerized-task, and physiological outcomes were analysed using linear mixed-effects models. A separate model was estimated for each dependent variable. Group (three levels: WL, sham-Cog, and tDCS-Cog), Time (two levels: t0 and t1), and the Group × Time interaction were entered as fixed effects. Participant was included as a random intercept to account for repeated observations. Within-participant residual dependency was modeled using a first-order autoregressive covariance structure. Models were estimated without transformation of the dependent variables. The principal intervention test was the omnibus Group × Time interaction, which assessed whether pre-to-post change differed across the three conditions. Significant interactions were followed by simple effects of Time within each group (Cohen’s *d* was used as effect size estimate for significant simple effects). Omnibus fixed effects were evaluated using *F* tests. Estimated marginal means and confidence intervals are reported for statistically significant effects. Two-sided statistical significance was set at *α* = 0.05. Also, the outcomes of mixed-effects models have been checked against the false discovery rate by applying the Benjamini-Hochberg procedure (Benjamini and Hochberg, 1995), so to minimize the risk of reporting and interpreting results biased by potential multiple testing errors. Yet, at the light of the sample size of this pilot study, we cautiously acknowledge the exploratory nature of reported inferential analyses.

## 3 Results

### 3.1 Neuropsychological outcomes

Stroop interference time did not show significant effects of Group (*F*(2, 30) = 2.18, *p* = 0.131), Time (*F*(1, 30) = 3.35, *p* = 0.077), or Group × Time (*F*(2, 30) = 0.50, *p* = 0.613). For errors at the Stroop test, a significant main effect of Time emerged (*F*(1, 30) = 12.78, *p* = 0.001), whereas the Group × Time interaction was not significant (*F*(2, 30) = 0.33, *p* = 0.722). Across groups, scores decreased from t0 (*M* = 0.78, 95% CI [0.1, 1.5]) to t1 (*M* = −0.57, 95% CI [−1.3, 0.1]).

Raven’s Progressive Matrices showed a significant Group × Time interaction (*F*(2, 30) = 3.43, *p* = 0.046, see Figure 1a and Supplementary Figure SF1a). In tDCS-Cog, scores increased significantly from t0 (*M* = 32.8, 95% CI [29.7, 35.9]) to t1 (*M* = 36.2, 95% CI [33.9, 39.3]; *p* = 0.017; *d* = 0.67). Scores did not change significantly in sham-Cog, from t0 (*M* = 34.5, 95% CI [31.2, 37.7]) to t1 (*M* = 36.4, 95% CI [33.1, 39.6]; *p* = 0.183), or in WL, from t0 (*M* = 33.5, 95% CI [30.5, 36.5]) to t1 (*M* = 32.2, 95% CI [29.2, 35.2]; *p* = 0.304).

**Figure 1:**
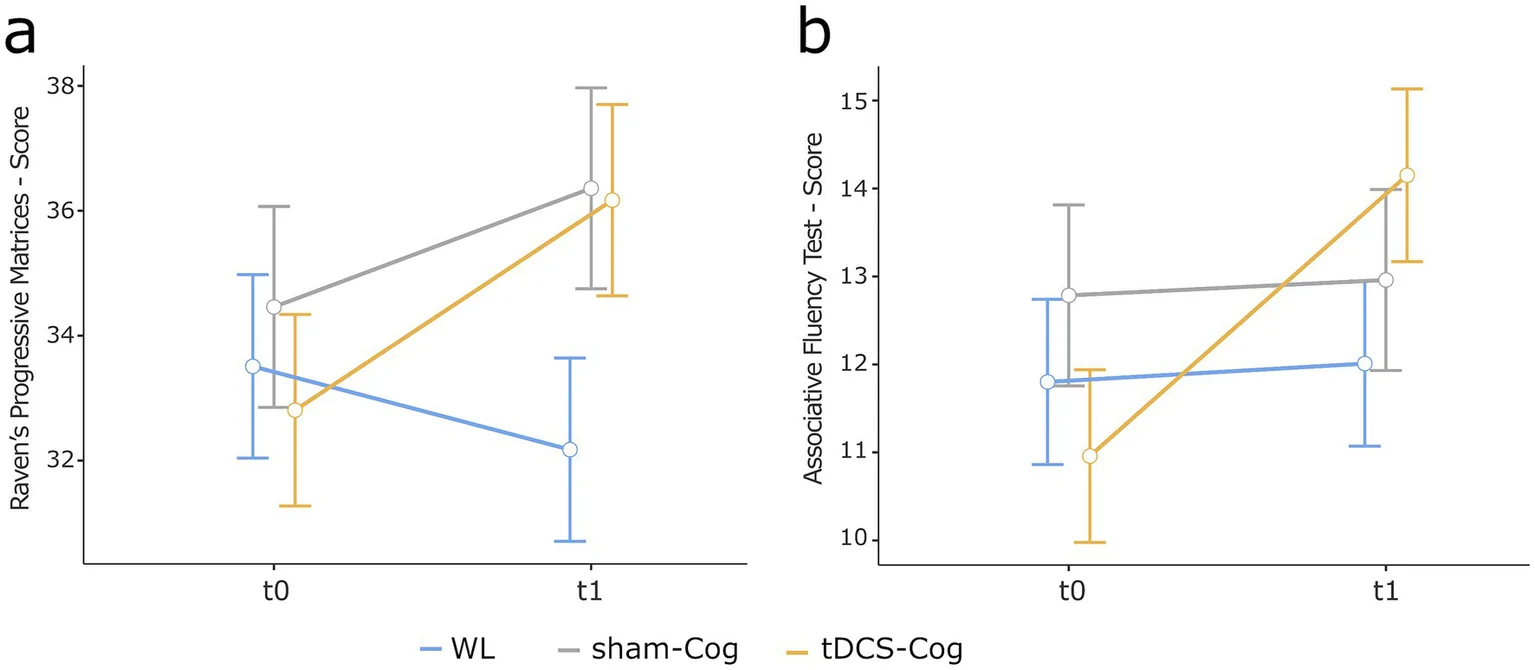
Comparison between wait-list (WL), sham-tDCS plus cognitive-training (sham-Cog), and active-tDCS plus cognitive-training (tDCS-Cog) group scores before (t0) and after (t1) training at: **(a)** Raven’s progressive matrices; **(b)** associative fluency test. Bars represent ±1 SE from the mean.

Phonemic fluency showed a significant main effect of Time (*F*(1, 30) = 4.84, *p* = 0.036), with scores increasing across groups from t0 (*M* = 37.3, 95% CI [33.5, 41.1]) to t1 (*M* = 39.6, 95% CI [35.8, 43.3]). The Group × Time interaction was not significant (*F*(2, 30) = 0.48, *p* = 0.621).

Category fluency also significantly increased depending on Time (*F*(1, 30) = 9.45, *p* = 0.004), from t0 (*M* = 22.7, 95% CI [21.3, 24.1]) to t1 (*M* = 24.0, 95% CI [22.6, 25.5]). The Group × Time interaction was not significant (*F*(2, 30) = 0.13, *p* = 0.877).

For associative fluency, significant effects emerged for Time (*F*(1, 30) = 9.38, *p* = 0.005), and Group × Time (*F*(2, 30) = 6.64, *p* = 0.004, see Figure 1b and Supplementary Figure SF1b). Scores increased significantly in tDCS-Cog from t0 (*M* = 11.0, 95% CI [9.0, 13.0]) to t1 (*M* = 14.1, 95% CI [12.2, 16.2]; *p* < 0.001; *d* = 0.95). No significant changes occurred in sham-Cog, from t0 (*M* = 12.8, 95% CI [10.7, 14.9]) to t1 (*M* = 13.0, 95% CI [10.9, 15.1]; *p* = 0.806), or in WL, from t0 (*M* = 11.8, 95% CI [9.9, 13.7]) to t1 (*M* = 12.0, 95% CI [10.1, 13.9]; *p* = 0.748).

For the Trail Making Test difference score, a significant main effect of Time emerged (*F*(1, 30) = 7.83, *p* = 0.009), but the Group × Time interaction was not significant (*F*(2, 30) = 1.11, *p* = 0.344). Across groups, scores decreased from t0 (*M* = 39.7, 95% CI [26.5, 52.8]) to t1 (*M* = 22.5, 95% CI [9.4, 35.6]), indicating faster performance.

Immediate verbal learning showed a significant main effect of Time (*F*(1, 30) = 43.13, *p* < 0.001), but no Group × Time interaction (*F*(2, 30) = 0.70, *p* = 0.504). Scores increased across groups from t0 (*M* = 49.1, 95% CI [46.0, 52.2]) to t1 (*M* = 55.9, 95% CI [52.8, 59.0]).

Delayed verbal recall also showed a significant main effect of Time (*F*(1, 30) = 15.65, *p* < 0.001), without a Group × Time interaction (*F*(2, 30) = 0.49, *p* = 0.615). Across groups, scores increased from t0 (*M* = 11.4, 95% CI [10.4, 12.5]) to t1 (*M* = 13.0, 95% CI [11.9, 14.0]).

Attention Matrices, verbal and visuospatial short-term memory, and visuospatial supra-span learning did not show significant Group, Time or Group × Time effects.

Estimated marginal means and 95% confidence intervals for all neuropsychological and computerized-task outcomes across groups and assessment time points, together with the corresponding inferential statistics, are reported in Supplementary Table S1.

### 3.2 Computerized attentional-task performance

Accuracy showed a significant main effect of Time (*F*(1, 25) = 16.98, *p* < 0.001), but no Group × Time interaction (*F*(2, 25) = 1.35, *p* = 0.277). Across groups, accuracy increased from t0 (*M* = 81.9%, 95% CI [77.8, 86.0]) to t1 (*M* = 88.7%, 95% CI [84.6, 92.8]).

Similarly, omissions showed a significant main effect of Time (*F*(1, 25) = 8.42, *p* = 0.008), but no Group × Time interaction (*F*(2, 25) = 0.89, *p* = 0.422). Across groups, omissions decreased from t0 (*M* = 10.01, 95% CI [7.0, 13.0]) to t1 (*M* = 5.04, 95% CI [2.0, 8.1]).

### 3.3 Physiological marker: N2 ERP

No significant Group × Time interactions emerged for N2 latency at F3, Fz, or F4 in either congruent or incongruent trials.

A significant Group × Time interaction emerged for N2 amplitude at F4 during congruent trials (*F*(2, 25) = 3.84, *p* = 0.035, see Figure 2). In tDCS-Cog, N2 amplitude became significantly more negative from t0 (*M* = −5.00, 95% CI [−7.1, −2.9]) to t1 (*M* = −9.12, 95% CI [−11.3, −7.0]; *p* = 0.003, *d* = 1.19). No significant change occurred in sham-Cog, from t0 (*M* = −4.57, 95% CI [−6.7, −2.4]) to t1 (*M* = −5.90, 95% CI [−8.0, −3.8]; *p* = 0.289), or in WL, from t0 (*M* = −4.77, 95% CI [−6.8, −2.7]) to t1 (*M* = −4.21, 95% CI [−6.3, −2.2]; *p* = 0.639).

**Figure 2:**
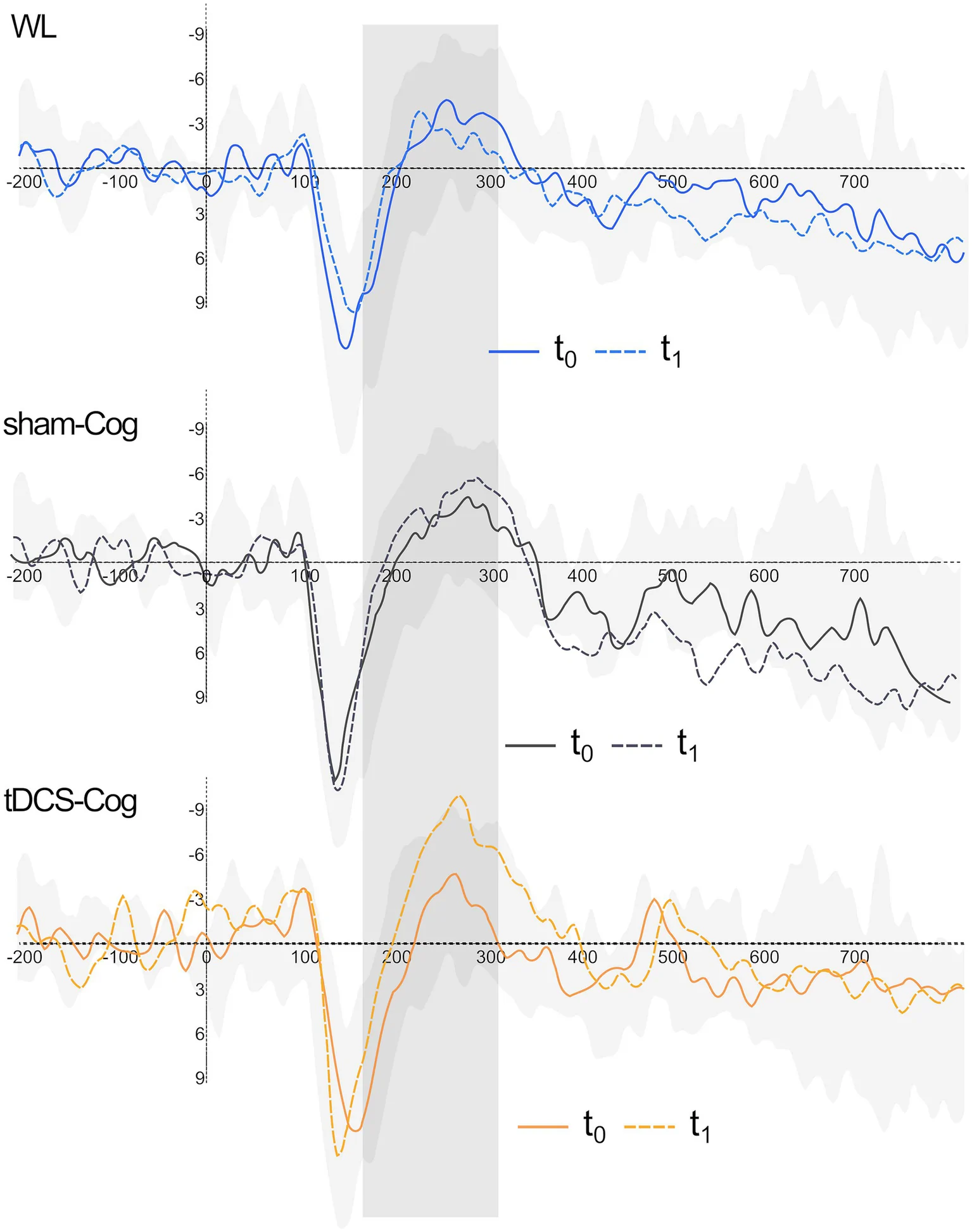
Graphical depiction of group average waveforms for wait-list (WL), sham-tDCS plus cognitive-training (sham-Cog), and active-tDCS plus cognitive-training (tDCS-Cog) groups before (t0) and after (t1) training, congruent stimuli of the Stroop-like computerized task (electrode: F4). Shaded areas represent ±1 SD from the group average waveforms at t0. The gray bar highlights the predefined time window within which individual N2 peak amplitudes were identified and extracted for statistical analysis.

The Group × Time interaction effect for N2 amplitude in incongruent trials at F4 approached but did not reach significance (*F*(2, 25) = 3.16, *p* = 0.060). Descriptively, in tDCS-Cog, N2 amplitude became more negative from t0 (*M* = −4.49, 95% CI [−6.7, −2.3]) to t1 (*M* = −7.72, 95% CI [−9.9, −5.5]). Conversely, N2 amplitude showed no relevant modulation in sham-Cog (t0: *M* = −5.72, 95% CI [−7.9, −3.5]; t1: *M* = −5.74, 95% CI [−7.9, −3.5]), or in WL (t0: *M* = −5.23, 95% CI [−7.3, −3.2]; t1: *M* = −4.56, 95% CI [−6.6, −2.5]).

No significant Group, Time, or Group × Time interactions emerged for N2 amplitude at F3 or Fz during congruent or incongruent trials.

## 4 Discussion

The present pilot study examined behavioral and electrophysiological changes following individualized cognitive training paired with either active or sham right-prefrontal tDCS, using a multimodal framework integrating neuropsychological performance with physiological markers of task-related processing. Overall, repeated active tDCS combined with cognitive training was associated with selective improvements in higher-order executive functioning and localized modulation of the right-prefrontal N2 ERP. By contrast, other cognitive outcomes improved over time without group differences, suggesting broader training or practice effects rather than changes specifically attributable to active stimulation.

The clearest intervention-specific behavioral findings concerned non-verbal reasoning and associative verbal fluency. Raven’s Progressive Matrices showed a significant Group × Time interaction, with pre-to-post improvement in tDCS-Cog but not in sham-Cog or WL. Performance requires logical reasoning, rule maintenance, hypothesis generation, relational integration, and monitoring of alternative solutions, supported by distributed frontoparietal networks and lateral prefrontal regions (Christoff et al., 2001; Jung and Haier, 2007). The observed pattern is therefore compatible with the hypothesis that repeated anodal stimulation over the right prefrontal cortex increased the responsivity of prefrontal systems engaged during cognitive training. Rather than producing generalized enhancement, active tDCS may have preferentially facilitated relational integration and strategic executive control.

A comparable pattern emerged for associative fluency. Performance improved substantially in tDCS-Cog, whereas sham-Cog and WL remained stable. Associative fluency requires flexible generation of semantically related responses without reliance on a single overlearned retrieval rule, placing demands on strategic search, monitoring, switching among semantic representations, and inhibition of repetitive or dominant responses. The Group × Time effect may indicate that active right-prefrontal stimulation enhanced the flexible regulation of internally generated response sets. Together, the Raven and associative-fluency findings suggest that active tDCS preferentially supported higher-order executive processes rather than elementary processing speed or memory storage.

This interpretation is consistent with activity-dependent accounts of tDCS. Weak direct current does not directly elicit neuronal firing but modifies membrane polarization and the probability that task-engaged neural populations will respond to ongoing input (Antal et al., 2017; Fertonani and Miniussi, 2017; Nitsche and Paulus, 2011; Siegert et al., 2021). In the present protocol, cognitive exercises began 5 min after stimulation onset, overlapped with the remaining stimulation period, and continued thereafter. This arrangement may have promoted concurrent engagement and modulation of right-prefrontal networks, potentially strengthening training-related plasticity. The repeated administration of 24 sessions may also have supported cumulative effects unlikely to emerge after a single session.

Nonetheless, several outcomes showed significant main effects of Time without Group × Time interactions. Measures of attention regulation, phonemic and category fluency, and immediate and delayed verbal learning improved across the sample. In the trained groups, some changes may reflect repeated adaptive exercises; however, improvements were also partly observed in WL. Repeated exposure, greater familiarity with testing procedures, and procedural learning therefore remain plausible contributors (Calamia et al., 2012). The absence of significant effects for Stroop interference time, Attention Matrices, and short-term memory further indicates that the intervention did not produce generalized enhancement. This selectivity accords with the broader NIBS literature, in which outcomes vary according to stimulation target, montage, dosage, baseline performance, task demands, and correspondence between the stimulated network and trained process (Antal et al., 2017; Indahlastari et al., 2021; Santarnecchi et al., 2015; Shin et al., 2015; Siegert et al., 2021; Summers et al., 2016). The findings therefore favor a process-specific rather than global account of neuroempowerment.

The electrophysiological results provide particularly relevant evidence for intervention specificity. Active tDCS combined with cognitive training produced a significant increase in N2 amplitude at F4, whereas neither sham-Cog nor WL changed significantly. This spatial localization is notable because F4 corresponded to the anodal stimulation site. Although scalp-recorded activity cannot be attributed uniquely to the underlying right prefrontal cortex, the topographical correspondence between the stimulation site and the significant electrophysiological modulation is compatible with preferential effects on right-frontal processing.

This finding also illustrates the value of physiological outcomes in NIBS studies. Behavioral measures indicate whether performance changes but provide limited information about where and when stimulation-related modulation occurs. ERPs can reveal spatially and temporally circumscribed effects, even when broader behavioral outcomes remain unchanged. Here, the significant N2 modulation observed at F4, corresponding to the anodal stimulation site, suggests a topographically selective pattern of intervention-related neurophysiological modulation. However, the nonsignificant effects at F3 and Fz should not be interpreted as evidence of absent modulation at these sites, particularly given the limited statistical power of the present pilot sample. Accordingly, the observed pattern is consistent with, but does not establish, preferential modulation of right-frontal processing. Physiological measures may therefore help determine whether behavioral gains are accompanied by modulation of the targeted network rather than nonspecific changes in arousal, motivation, or task familiarity.

The N2 has been associated with conflict detection, mismatch processing, response selection, salience evaluation, and allocation of cognitive-control resources (Folstein and Van Petten, 2008; Larson et al., 2014). Greater N2 negativity following active stimulation may therefore reflect enhanced recruitment of right-prefrontal resources during early stimulus evaluation. Within aging frameworks, such increased recruitment could potentially reflect compensatory upregulation, provided that additional recruitment supports performance rather than reflecting neural inefficiency (Cabeza et al., 2018), but increased neural activity cannot in itself distinguish adaptive recruitment from neural inefficiency. Although increased right-prefrontal N2 negativity and improved executive outcomes co-occurred in tDCS-Cog, their association was not directly tested; therefore, the functional significance of the N2 modulation remains to be established.

The restriction of the significant N2 effect to congruent trials may be interpreted in light of stimulus frequency. Congruent stimuli were less frequent than incongruent stimuli and may therefore have acquired greater contextual relevance and perceptual salience. Rare events generally require stronger updating of the current stimulus representation and may recruit additional attentional resources because they violate the prevailing task context. Accordingly, the localized N2 enhancement may reflect increased sensitivity of the stimulated right-prefrontal system to infrequent, salient, or contextually unexpected events rather than modulation of conflict processing alone. This interpretation is compatible with a broader role of the N2 in salience detection, mismatch evaluation, and adaptive attentional allocation (Carretié et al., 2004; Folstein and Van Petten, 2008; Larson et al., 2014; Mishra et al., 2015).

The Group × Time interaction for N2 amplitude at F4 during incongruent trials also approached significance and showed a descriptively similar increase in negativity in tDCS-Cog. This suggests that modulation may not have been entirely restricted to the infrequent congruent condition. However, because the interaction did not reach conventional significance, this interpretation remains speculative.

No intervention-related effects emerged for N2 latency or amplitude at F3 or Fz. The absence of significant latency modulation suggests that the present analyses did not detect intervention-related changes in the timing of early stimulus evaluation. Although the topographical amplitude pattern is compatible with preferential modulation near the intended stimulation target, the nonsignificant findings at F3 and Fz cannot establish the absence of effects at these sites. Therefore, the present results suggest a potentially spatially selective pattern, rather than demonstrating anatomically circumscribed modulation.

The convergence between selective behavioral and electrophysiological findings is theoretically relevant. Active intervention modulated higher-order executive outcomes and right-prefrontal N2 amplitude while leaving several other measures unchanged. This is compatible with a mechanism in which tDCS amplified the engagement of right-prefrontal networks repeatedly recruited during training. More broadly, it shows how physiological outcomes can clarify whether behavioral changes are accompanied by modulation of the expected neural system. Future studies should collect electrophysiological activity during a wider range of cognitive tasks and test whether individual neural changes predict behavioral improvement. Such analyses could distinguish responders from non-responders, determine whether increased recruitment reflects adaptive compensation or inefficient overactivation, and assess whether stimulation effects generalize across tasks engaging the same network. High-density EEG and source reconstruction would further clarify whether scalp-level effects correspond to the intended cortical target and connected frontoparietal networks.

The findings can be situated within models of neural maintenance, reserve, and compensation in aging. Older adults frequently recruit additional prefrontal resources to support performance, as described by the HAROLD and PASA frameworks (Cabeza, 2002; Davis et al., 2008). Repeated right-prefrontal stimulation may have strengthened the capacity to mobilize such resources during demanding operations. However, increased activity cannot be regarded as compensatory solely on the basis of magnitude. Multimodal evidence is required to determine whether physiological modulation is topographically appropriate, behaviorally beneficial, and sustained over time.

In addition, we acknowledge the difficulty of fully disentangling stimulation-specific effects from other factors inherent to an integrated intervention. Although the sham-tDCS plus cognitive-training condition provided control for the cognitive-training program and for stimulation-related expectancy and procedural factors, participants in both intervention groups were exposed to a structured training context involving repeated researcher contact, sustained attention, task engagement, and other contextual or relational factors associated with participation in an intervention. The wait-list condition controlled more broadly for changes related to repeated assessment and the passage of time, but did not account for these nonspecific intervention-related factors. Accordingly, the present design is best interpreted as testing the additional contribution of active tDCS when delivered in conjunction with cognitive training, rather than isolating a pure stimulation effect. The selective changes observed in the active-tDCS condition, particularly at the electrophysiological level, are therefore consistent with a stimulation-related contribution but cannot be considered independent of the broader intervention context. Future factorial designs including cognitive-training-only, tDCS-only, sham, and no-intervention conditions would allow the independent and interactive contributions of training, stimulation, and intervention-related contextual factors to be more directly examined.

Several further limitations should be also acknowledged. First, the small sample limits statistical power and precision and increases sensitivity to interindividual differences in cortical anatomy, baseline performance, and stimulation responsiveness. Second, the EEG montage and peak-based ERP analysis provided limited spatial resolution. Indeed, F4 activity cannot be uniquely attributed to the right dlPFC because ERP signals reflect volume-conducted activity. Larger studies using high-density EEG and source reconstruction would better characterize the affected networks. Furthermore, because stimulus congruency and frequency were not independently manipulated, the present design does not allow the contribution of congruency per se to be disentangled from that of stimulus rarity in explaining the condition-specific N2 modulation. Condition-specific behavioral accuracy was also unavailable, preventing direct examination of whether the differential electrophysiological pattern was paralleled by condition-specific performance changes. Finally, the study focused on immediate pre-to-post change and therefore does not establish the persistence of the observed behavioral and electrophysiological effects. The present findings should consequently be interpreted as evidence of intervention-related modulation rather than stable neuroplastic adaptation. Longitudinal follow-up will be necessary to determine whether these changes persist after the end of training and can therefore be considered indicative of more durable neuroplastic reorganization.

## 5 Conclusion

The present pilot study provides preliminary multimodal evidence that repeated anodal tDCS over the right prefrontal cortex, delivered concurrently with individualized cognitive training, may selectively support executive functioning in healthy older adults. The intervention-specific behavioral effects on non-verbal reasoning and associative fluency were accompanied by increased N2 negativity over the right-prefrontal stimulation site. The topographical correspondence between the significant physiological effect at F4 and the anodal electrode position is compatible with preferential right-frontal modulation, although the present montage does not allow the contribution of individual electrodes or affected cortical networks to be disentangled. These findings illustrate the added value of physiological outcome measures for determining whether NIBS-related effects involve the intended neural system and for distinguishing spatially and functionally specific neurophysiological modulation from nonspecific behavioral change.

Current findings, although exploratory, suggest that NIBS-supported neuroempowerment may operate by strengthening specific task-relevant prefrontal processes rather than producing broad cognitive enhancement. Integrating behavioural measures with temporally and spatially sensitive physiological markers may therefore be essential for developing mechanism-guided stimulation protocols, characterizing potentially adaptive versus inefficient neural responses, and clarifying how stimulation-site specificity contributes to treatment efficacy.

## Data Availability

The raw data supporting the conclusions of this article will be made available by the authors, without undue reservation.
